# Activation of NRF2 Signaling Pathway Delays the Progression of Hyperuricemic Nephropathy by Reducing Oxidative Stress

**DOI:** 10.3390/antiox12051022

**Published:** 2023-04-28

**Authors:** Panshuang Qiao, Yi Sun, Yiming Wang, Simei Lin, Yongpan An, Liang Wang, Jihan Liu, Yajun Huang, Baoxue Yang, Hong Zhou

**Affiliations:** 1 State Key Laboratory of Natural and Biomimetic Drugs, Department of Pharmacology, School of Basic Medical Sciences, Peking University, Beijing 100191, China; panshuangqiao@bjmu.edu.cn (P.Q.); wangyiming1211@pku.edu.cn (Y.W.); linsimei@pku.edu.cn (S.L.); anyongpan123@bjmu.edu.cn (Y.A.); wangliang94@bjmu.edu.cn (L.W.); 2211210032@stu.pku.edu.cn (J.L.); yajun@pku.edu.cn (Y.H.); baoxue@bjmu.edu.cn (B.Y.); 2 Department of Pharmacology, School of Pharmacy, China Pharmaceutical University, Nanjing 210009, China; 1020162522@cpu.edu.cn; 3 Department of the Integration of Chinese and Western Medicine, School of Basic Medical Sciences, Peking University, Beijing 100191, China

**Keywords:** hyperuricemic nephropathy, NRF2, oxidative stress, mitochondria, chronic kidney disease, tubulointerstitial fibrosis

## Abstract

Hyperuricemia (HUA)-induced oxidative stress is a crucial contributor to hyperuricemic nephropathy (HN), but the molecular mechanisms underlying the disturbed redox homeostasis in kidneys remain elusive. Using RNA sequencing, together with biochemical analyses, we found that nuclear factor erythroid 2-related factor 2 (NRF2) expression and nuclear localization levels were increased in early HN progression and then gradually declined below the baseline level. We identified the impaired activity of the NRF2-activated antioxidant pathway as a driver of oxidative damage in HN progression. Through *nrf2* deletion, we further confirmed aggravated kidney damage in *nrf2* knockout HN mice compared with HN mice. In contrast, the pharmacological agonist of NRF2 improved kidney function and alleviated renal fibrosis in mice. Mechanistically, the activation of NRF2 signaling reduced oxidative stress by restoring mitochondrial homeostasis and reducing NADPH oxidase 4 (NOX4) expression in vivo or in vitro. Moreover, the activation of NRF2 promoted the expression levels of heme oxygenase 1 (HO-1) and quinone oxidoreductase 1 (NQO1) and enhanced the antioxidant capacity of cells. Furthermore, the activation of NRF2 ameliorated renal fibrosis in HN mice through the downregulation of the transforming growth factor-beta 1 (TGF-β1) signaling pathway and ultimately delayed the progression of HN. Collectively, these results suggested NRF2 as a key regulator in improving mitochondrial homeostasis and fibrosis in renal tubular cells by reducing oxidative stress, upregulating the antioxidant signaling pathway, and downregulating the TGF-β1 signaling pathway. The activation of NRF2 represents a promising strategy to restore redox homeostasis and combat HN.

## 1. Introduction

Hyperuricemia (HUA) is classically defined as an elevation of serum uric acid (SUA) levels beyond the solubility threshold, which is caused by uric acid (UA) overproduction from hepatic metabolism and cell turnover, renal UA underexcretion, or extra-renal uric underexcretion, or both [1]. The prevalence of HUA in mainland China was 13.3% (19.4% in men and 7.9% in women), which was in accordance with the worldwide prevalence rate reported to range from 2.6% to 36% in different populations [2,3]. Although HUA is considered to be the most important risk factor for gout, only approximately 10% of patients with HUA will eventually experience a gout attack [4]. Thus, genetic factors appear to play a more prominent role in gout than in other diseases described below [4].

Recent studies have reported that HUA is a potential risk factor for developing insulin resistance, lipid abnormalities, and hypertension. Therefore, HUA is found to be significantly associated with numerous comorbidities, such as metabolic syndrome (MetS), obesity, type 2 diabetes, renal disease, and cardiovascular diseases (CVDs), including hypertension, atherosclerosis, and heart failure. However, the molecular mechanism of how HUA affects these metabolic diseases, kidney diseases, or CVDs is unclear [5,6]. Furthermore, in certain preclinical and clinical studies, there remains an argument about whether xanthine oxidase (XO) inhibitors can improve these diseases.

As one common clinical complication of HUA, hyperuricemic nephropathy (HN) is a common cause of acute kidney injury (AKI) [7]. Moreover, increasing evidence indicates that an abnormally high SUA level contributes to the chronic decline of renal function and is an independent risk factor of chronic kidney disease (CKD) [8,9]. CKD is a global public health problem with high morbidity and mortality, and it is also accompanied by profound HUA as UA clearance declines with CKD progression [10,11,12,13]. In CKD experimental models, continuously high levels of SUA led to endothelial dysfunction, oxidative stress, tubular injury, and systemic and local inflammation in the kidney [14]. Although several genes, cytokines, and signaling cascades have been reported to be involved in the development of HUA-induced HN and CKD, the mechanism from HN to CKD remains unknown. Additionally, a subject of debate concerning whether it is effective to delay the progression of CKD by using urate-lowering therapy in patients with asymptomatic HUA or CKD still remains [15,16].

As the organ with the second largest mitochondrial contents in the human body, whose main sources of reactive oxygen species (ROS) in renal tubular cells are the mitochondrial oxidative respiratory chain and NADPH oxidase 4 (NOX4), the kidney is extremely vulnerable to damage caused by oxidative stress [17]. Oxidative stress is now recognized as a major pathogenic factor for various progressive clinical and experimental renal diseases [17]. ROS is intimately involved in cell signaling, and the dysregulation of antioxidant mechanisms and the overproduction of ROS leads to mitochondrial dysfunction and further exacerbates kidney injury [18].

Recently, nuclear factor erythroid 2-related factor 2 (NRF2) has been recognized as a master regulator against cellular oxidative stress, which exerts its antioxidant function mainly through the transcriptional activation of cytoprotective gene expression [19]. The activation of NRF2 exerts protective effects by ameliorating mitochondrial dysfunction, oxidative damage, inflammation, and fibrosis in various renal diseases, including AKI, CKD, and diabetic nephropathy, which highlights NRF2 antioxidant pathway as an important target for kidney disease treatment [20,21]. However, it is not clear whether NRF2 plays an important role in HN development.

Therefore, this study focuses on the role of NRF2 in HN progression and whether the activation of the NRF2-dependent antioxidative pathway would be a promising strategy to alleviate HN. Our results, for the first time, provide evidence that activating NRF2 improves mitochondrial homeostasis by reducing oxidative stress and upregulating antioxidant signaling pathways ultimately alleviates renal fibrosis by downregulating transforming growth factor-beta 1 (TGF-β1) signaling pathways.

## 2. Materials and Methods

### 2.1. RNA Sequencing

RNA was extracted from 100 mg of the kidney cortex of healthy mice and HN mice, respectively. Three biological replicates were used for each group. Total RNA was extracted using the RNeasy Plus Kit (Qiagen, Hilden, Germany) and RNA concentration was determined using the NanoDrop 2000 (Thermo Fisher Scientific, Waltham, MA, USA) according to the manufacturer’s instructions. RNA-Seq, data generation, and normalization were performed by Majorbio Bio-Pharm Technology Co., Ltd. (Shanghai, China). Details about the genomic data are summarized in the Supplementary Materials, S2.

### 2.2. Bioinformatics Analysis of Renal Tubular Samples from Normal Individuals or Patients with CKD

Data on the distribution of NRF2 in the whole kidney were obtained from the gene expression profiling of different kidney structures in 34 normal kidney samples. Data on NRF2 expression in renal tubules were obtained from the gene expression profiles of microscopically dissected tubulointerstitial samples from a discovery cohort of 170 European renal cDNA Biobank (ERCB) CKD patients and 31 healthy living donors. Patients with different types of renal disease were confirmed by renal biopsy. All clinical data from normal subjects and CKD patients can be downloaded from the Nephroseq database of open-access human microarray datasets of published studies (https://www.nephroseq.org/resource/main.html, accessed on 24 February 2023).

### 2.3. Cell Culture and Cell Models

The NRK-52E cells (rat renal proximal tubule epithelial cells) were purchased from the Cell Resource Center of Shanghai Institutes for Biological Sciences, Chinese Academy of Sciences (Shanghai, China). NRK-52E cells were cultured in Dulbecco’s modified Eagle’s medium (DMEM) containing 10% fetal bovine serum (FBS; Gibco, Melbourne, Australia), 1% non-essential amino acids, 1% sodium pyruvate, 100 U/mL penicillin, and 100 μg/mL streptomycin in a humidified atmosphere with 5% CO_2_ at 37 °C.

Cells were seeded in 6-well plates. When cells reached 40–50% confluency, cells were incubated with 8 mg/dL UA for 24 h in the absence or presence of 0.5 μmol/L SFN. Finally, the cells are harvested for subsequent testing.

### 2.4. Animal Experiments and Ethics

Healthy 8~9-week-old male C57BL/6J mice weighing 20 g to 23 g were purchased from the Animal Center of Peking University Health Science Center (Beijing, China). *Nrf2* knockout (KO) mice (B6.129 × 1-Nfe2l2tm1Ywk/J) were obtained from the Jackson laboratory [22]. Mice were housed in open-top conventional cages with poplar bedding material in a pathogen-free environment. All mice were exposed to a 12 h light/dark cycle under defined environmental conditions at 25 ± 2 °C with a relative humidity of 50% and free access to food and water. All procedures in this study were carried out in strict accordance with the recommendations in the Guide for the Care and Use of Laboratory Animals of China Association for Laboratory Animal Science. All animal care and protocols were approved by the Institutional Animal Care and Use Committee at the Peking University Health Science Center (Beijing, China, LA220354, 19 May 2020). All sacrifice was performed under anesthesia with pentobarbitone.

Eight-week-old male C57BL/6J mice were randomly divided into 6 groups (6 mice in each group): control group, HUA progress 1 day group, HUA progress 3 days group, HUA progress 5 days group, HUA progress 7 days group, and HUA progress 14 days group. The HUA mouse model was induced via the intraperitoneal injection of a mixture of 300 mg/kg potassium oxonate and 300 mg/kg hypoxanthine for 1, 3, 5, 7, and 14 days, respectively, to simulate the occurrence and progression of HUA in mice. The control group was intraperitoneally injected with the same amount of normal saline.

Eight-week-old male C57BL/6J mice were randomly divided into 5 groups (6 mice in each group): control group, control administration group (2.5 mg/kg SFN), HN model group, low dose group (0.5 mg/kg SFN), and high dose group (2.5 mg/kg SFN). The HN mouse model was induced via the intraperitoneal injection of a mixture of 300 mg/kg potassium oxonate and 300 mg/kg hypoxanthine for 14 consecutive days. The control group and the model group were given NRF2 agonist SFN by subcutaneous injection, and the control group and model group were given the same amount of sterile solvent by subcutaneous injection for 14 consecutive days.

KO and wild-type littermates (WT) were divided into four groups, 6 mice in each group: WT control group, KO control group, WT model group, and KO model group. The HN mouse model was induced in the WT model group and the KO model group via the intraperitoneal injection of a mixture of 300 mg/kg potassium oxonate and 150 mg/kg hypoxanthine for 3 consecutive days. The WT control group and the KO control group were intraperitoneally injected with the same amount of normal saline.

### 2.5. Analysis of Intracellular ROS

Cells were plated in 6-well plates and incubated with 8 mg/dL of UA in the absence or presence of 0.5 μmol/L of sulforaphane (SFN) (MCE, Shanghai, China, Cat# HY-13755) for 24 h. Cells were then harvested and stained with dichlorodihydrofluorescein diacetate (DCFH-DA; Beyotime, Nanjing, China, Cat# S0033S) according to the manufacturer’s protocol. Each sample was examined by fluorescence microscopy.

### 2.6. Determination of Adenosine Triphosphate (ATP) Content

Kidney tissue and NRK-52E cell ATP content were determined via an Enhanced ATP Assay Kit (Beyotime, Nanjing, China, Cat# S0027), according to the manufacturer’s instructions.

### 2.7. Mitochondrial Membrane Potential

Cells were cultured on six-well plates and the medium was replaced with 10 μg/mL JC-1 (dissolved in DMSO) containing an 1× Assay buffer (JC-1 Mitochondrial Membrane Potential Detection Kit, Beijing Solarbio Science & Technology Co., Ltd., Beijing, China, Cat# M8650). The cells were placed back into the incubator (37 °C, 5% CO_2_) for 20 min, followed by washing with phosphate-buffered saline (PBS) (2 times) to remove unbound dye. Monomeric JC-1 was detected via excitation at 488 nm and emission at 527 nm. Aggregated JC-1 was detected by excitation at 543 nm and emission at 570 nm using a fluorescent microscope (Nikon, Tokyo, Japan, ECLIPSE Ti2-U). Fluorescence intensity was measured using image-Pro plus 6.0 software (National Institutes of Health, Bethesda, MD, USA).

### 2.8. Biochemical Analysis of Serum/Urine/Kidney Samples

SUA (Cat# C012-2), UA in urine (Cat# C012-2), serum creatinine (Scr) (Cat# C011-2-1), and blood urea nitrogen (BUN) (Cat# C013-2-1) were measured using commercial assay kits (NJJC Bio, Nanjing, China), according to the manufacturer’s instructions. The H_2_O_2_ (Cat# A064-1-1), glutathione (GSH) (Cat# A006-2-1) level, superoxide dismutase (SOD) (Cat# A001-3-2) enzyme activity, and glutathione peroxidase (GSH-Px) enzyme activity (Cat# A005-1-1) in the mouse kidney were detected using commercial assay kits (NJJC Bio, Nanjing, China), according to the manufacturer’s instructions.

### 2.9. Non-Invasive Transcutaneous Assessment of Glomerular Filtration Rate (GFR)

Under light anesthesia (2% sevoflurane), a part of the mouse’s back was depilated. A transdermal reader-sensor device (MediBeacon, Mannheim, Germany) was attached to the skin in the dorsal region using a double-sided patch (MediBeacon, Mannheim, Germany). Fluorescein-labeled sinistrin tracer (FITC-S-Fresenius Kabi, Austria) was injected via the tail vein at a dose of 7.5 mg/100 g body weight. Mice were kept in individual cages for 1 h. The elimination kinetics curve for FITC-S gives individual transcutaneous glomerular filtration rate (tGFR) values. Data were analyzed in 3 sections using MB Studio (MediBeacon, Germany). All animals displayed repeated measurements at different time points but always from the same sensor set [23].

### 2.10. Histological Analysis of Kidney

The kidneys were fixed with 4% paraformaldehyde overnight, dehydrated in graded alcohol, and then embedded in paraffin for staining with hematoxylin-eosin (H&E) and Masson staining to assess the level of renal injury and collagen deposition. Images were captured using a Nikon confocal microscope.

### 2.11. Transmission Electron Microscopy

The tissues were transferred to an EP tube containing 2.5% glutaraldehyde and fixed at 4 °C for more than 24 h and then washed three times with 0.1M PBS for 15 min each time. Specimens were then fixed with 1% osmium acid at room temperature for 2 h in the dark. Next, the tissues were dehydrated with 30%, 50%, 70%, 80%, 95%, 100%, and 100% alcohol for 20 min each time and 100% acetone twice for 15 min each time. After dehydration, the sections were embedded in 812 resin overnight at 37 °C using acetone as a transitional solvent. The resin blocks were sliced in an ultra-thin slicing machine to 60–80 nm, stained on a 150-mesh copper mesh, and dried overnight. Finally, the mitochondria of proximal tubule cells were observed under a transmission electron microscope, and the images were collected and analyzed.

### 2.12. Immunohistochemistry

The kidney specimens were fixed in 4% paraformaldehyde overnight and embedded in paraffin. Tissue microarray sections were blocked with 5% bovine serum albumin for 1 h and then incubated with the indicated primary antibodies against NRF2 (ABclonal, Wuhan, China, Cat# A0674, 1:200 dilution) and α-SMA (Proteintech, Wuhan, China, Cat# 14395-1-AP, 1:1000 dilution) overnight. After incubation, kidney sections were incubated with Cy3-conjugated Affinipure goat anti-rabbit IgG (H + L) (Proteintech, Wuhan, China, Cat# SA00009-2, 1:1000 dilution) secondary antibodies followed by staining with the 3,3′diaminobenzidine substrate. DAB (ZSGB-BIO, Beijing, China, Cat# ZLI-9017) was used for antibody reactions. The non-immune goat IgG was used as the negative control. The slides were counterstained with hematoxylin, then dehydrated and mounted. Immunohistochemistry sections were imaged using a Nikon microscope.

### 2.13. Immunofluorescence Staining

Kidneys were fixed by 4% paraformaldehyde, gradient dehydrated by alcohol (Tong Guang, Beijing, China, Cat# 104022), and embedded in paraffin (Leica, Wetzlar, Germany, Cat# 39601095). Sections of 5 μm were cut, and 5% (*w*/*v*) bovine serum albumin was used to block the sections at room temperature for 1 h. Then, the sections were incubated with primary antibodies against NRF2 at 4 °C overnight. The next day, sections were washed 3 times and incubated with Cy3-conjugated Affinipure goat anti-rabbit IgG (H + L) secondary antibodies for 1 h. DAPI (Sigma, Saint Louis, MO, USA, Cat# D9542) was used to stain nuclei. Images were captured using a Nikon fluorescence microscope.

Cells were washed twice in PBS and blocked for 1 h in 5% (*w*/*v*) bovine serum albumin, and then the cells were incubated with the antibodies against NRF2 at 4 °C overnight. After primary antibody incubation, cells were washed 3 times and incubated with Cy3-conjugated affineur goat anti-rabbit IgG (H + L) secondary antibodies. DAPI (Sigma, Saint Louis, MO, USA, Cat# D9542) was used to label nuclei. Images were captured by a Nikon confocal microscope.

### 2.14. Western Blot Analysis

Take 20 mg of renal cortex from each group of mice and add 200 μL of RIPA lysis buffer (Applygen, Beijing, China, Cat# C1053) containing 4% protease inhibitor cocktail (Roche, South San Francisco, CA, USA, Cat# 1187358001) and 1% phosphatase inhibitor (Applygen, Beijing, China, Cat# P1260) to extract tissue protein.

Cells were seeded in 6-well plates and incubated with 8 mg/dL UA in the absence or presence of 0.5 μmol/L SFN for 24 h. Cell proteins were then extracted by adding 100 μL per well of the RIPA lysis buffer described above.

The protein concentration was detected by BCA Kit (Pierce, Rockford, IL, USA) after ultrasonic cracking. Identical amounts of protein samples were electrophoresed on polyacrylamide gels and electrotransferred to polyvinylidene difluoride membranes (PALL, Beijing, China, Cat# BSP0161). The membranes were blocked with blocking buffer (TBS, 0.1% Tween-20, 5% non-fat milk or 2% BSA) for 2 h at room temperature and incubated with primary antibodies against NOX4 (ABclonal, Wuhan, China, Cat# A11274, 1:1000 dilution), HO-1 (Proteintech, Wuhan, China, Cat# 10701-1-AP, 1:1000 dilution), NQO1 (Proteintech, Wuhan, China, Cat# 67240-1-Ig, 1:1000 dilution), MFN1 (ABclonal, Wuhan, China, Cat# A9880, 1:1000 dilution), MFN2 (ABclonal, Wuhan, China, Cat# A19678, 1:1000 dilution), FIS1 (ABclonal, Wuhan, China, Cat# A19666 1:1000 dilution), TGF-β1 (Proteintech, Wuhan, China, Cat# 21898-1-AP, 1:1000 dilution), α-SMA (Proteintech, Wuhan, China, Cat# 14395-1-AP, 1:5000 dilution), collagen 1 (Proteintech, Wuhan, China, Cat# 67288-1-Ig, 1:5000 dilution), and GAPDH (ABclonal, Wuhan, China, Cat# AC001, 1:10,000 dilution) at 4 °C overnight, respectively. Afterward, HRP-conjugated goat anti-rabbit IgG (H + L) (Immunoway, Suzhou, China, Cat# RS0002) or HRP-conjugated goat anti-mouse IgG (Easybio, Beijing, China, Cat# BE0102) were incubated for 60 min at room temperature. The ECL Kit (Meilunbio, Dalian, China, Cat# MA0186) was used to detect protein expression signal intensity through a chemiluminescence detection system (Syngene, Frederick, MD, USA, GeneGnome XRQ). Relative protein expression levels were quantified using ImageJ software (National Institutes of Health, Bethesda, MD, USA).

### 2.15. Statistical Analyses

All results were presented as means ± SEM. Statistical analyses were performed using GraphPad Prism 8.0 software (GraphPad Software Inc., La Jolla, CA, USA). For normally distributed continuous variables, an unpaired two-tailed Student’s *t*-test was used for statistical significance between two independent experimental groups, and one-way ANOVA followed by Tukey’s post hoc test was used for multiple group comparison. Correlations between data on mice were analyzed using linear regression analysis and the sample Pearson’s correlation coefficient. *p* values were determined using linear regression analysis. Immunofluorescent staining, immunohistochemical staining, and other fluorescent images were analyzed using open-source software (FIJI, VMTK, Stardist, MATLAB, DBSCAN). *p* < 0.05 was considered statistically significant.

## 3. Results

### 3.1. Renal Function Impairment in Hyperuricemic Mice Worsens with Disease Progression

**Figure 1:**
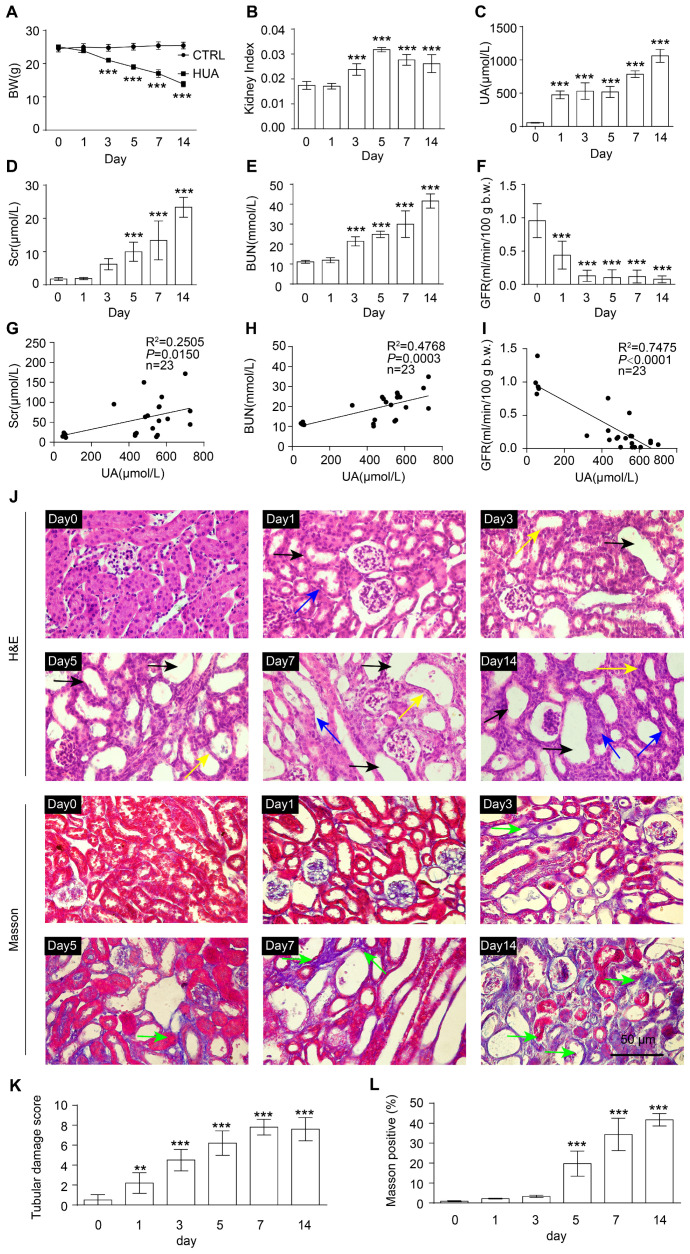
HUA progresses to HN with the aggravation of renal function injury. (**A**) Body weight (BW) in the indicated groups. (**B**) Kidney index (kidney weight–to–body weight ratios) in the indicated groups. (**C**) SUA level in the indicated groups. (**D**) Scr in the indicated groups. (**E**) BUN in the indicated groups. (**F**) GFR in the indicated groups. (**G**–**I**) Correlation between SUA with various parameters of kidney function: Scr (**G**), BUN (**H**), and GFR (**I**). (**J**) Hematoxylin and eosin (H&E, up) staining and Masson (down) staining of kidney tissue. Black arrows represent dilation, blue arrows represent atrophy, and yellow arrows represent loss of brush borders. Green arrows represent collagen deposits that are stained blue. Scale bar, 50 μm. (**K**) Quantification of tubular injury score in mouse kidney sections. (**L**) Quantification of Masson-stained positive areas in mouse kidney sections. Data represent means ± SEM. ** *p* < 0.01 and *** *p* < 0.001 vs. wild-type mice injected with inducer for 0 day. Data for each mouse are illustrated with the number of mice indicated in parentheses (**G**–**I**) or the data are from the kidneys of six mice (**A**–**F**, **J**–**L**).

To observe the process of occurrence and progression of HN, we continuously administered inducers for 0, 1, 3, 5, 7, and 14 days, respectively, to obtain wild-type HUA mice with different progressions (Figure S1A). With the extension of modeling days, the body weight of HUA mice was significantly reduced and the kidney index (kidney weight-to-body weight ratios) was significantly increased (Figure 1A,B). At baseline, SUA levels ranged between 45 and 65 μmol/L, which increased in HUA mice to 900–1200 μmol/L on the 14th day, whereas SUA levels of the control mice were maintained at the baseline (Figure 1C). As the disease progressed, the urine output of mice gradually increased, while the level of urine UA and urine osmolality gradually decreased (Figure S1B–D). Moreover, features of HN were displayed by HUA mice, indicated by a significant increase in Scr and BUN as well as a decline in GFR, compared with control mice without kidney impairment (Figure 1D–F).

We further explored the relationship between SUA levels and HN progression. It was found that SUA levels were positively correlated with Scr and BUN concentrations and negatively correlated with GFR, suggesting a significant relevance between SUA and renal function biomarkers in HN mice (Figure 1G–I).

The results of H&E staining also revealed the progressive histological abnormalities of HN mice, including proximal tubule dilation, atrophy, and the loss of brush borders in the renal cortex (Figure 1J,K). Masson staining showed increasingly serious collagen deposition in the kidneys of HN mice as the disease progressed (Figure 1J,L). In conclusion, the impairment of renal function and structure in HUA mice was aggravated with the development of the disease.

### 3.2. NRF2 Antioxidant Signaling Pathway Is Impaired in the Kidney of HN Mice

**Figure 2:**
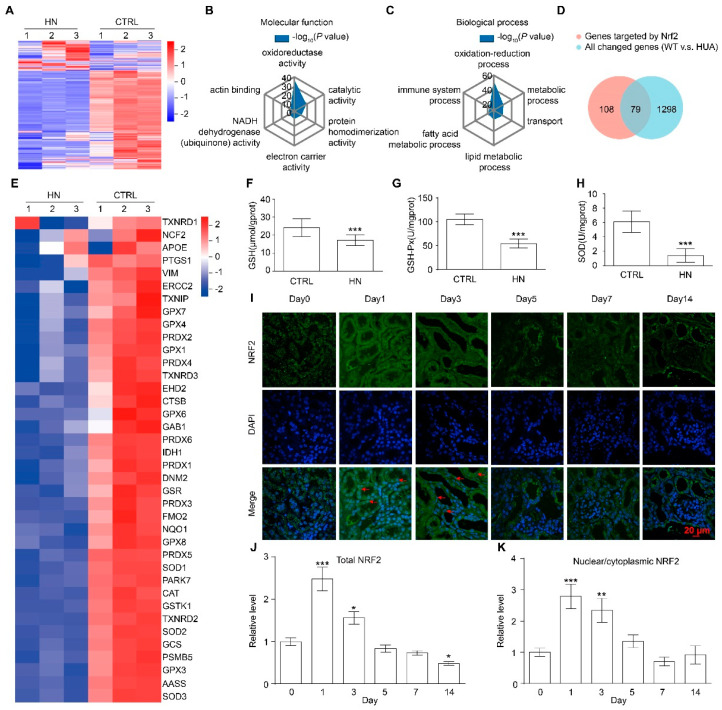
NRF2 signaling pathway is impaired in the kidney of HN mice. (**A**) Heatmap of gene abundance values from kidneys from CTRL and HN mice. RNA-sequencing analysis was performed with kidneys from three mice of each group. Data for each biological replicate are shown. (**B**,**C**) Gene ontology (GO) enrichment analysis of differentially expressed genes (DEGs). (**D**) Venn diagram from NRF2 target genes and DEGs. (**E**) Heatmap of the expression of 38 common NRF2 target genes in DEGs. (**F**) Glutathione (GSH) levels in kidneys in the indicated groups. (**G**) Glutathione peroxidase (GSH-PX) activity in kidneys in the indicated groups. (**H**) Superoxide dismutase (SOD) activity in kidneys in the indicated groups. (**I**) Immunofluorescence staining of NRF2 in kidneys in the indicated groups. Scale bar, 20 μm. Arrows indicate NRF2 nuclear localization. DAPI, 4’,6-diamidino-2-Phenyl indole. (**J**) Quantification of the expression of overall NRF2 in (**I**). (**K**) Quantification of the ratio of NRF2 in the nucleus to NRF2 in the cytoplasm in (**I**). Data represent means ± SEM. * *p* < 0.05, ** *p* < 0.01, and *** *p* < 0.001 vs. control (CTRL) mice. Data are from the kidneys of three (**A**–**E**) or six mice (**F**–**I**).

To identify the pathogenesis and the potential therapeutic targets for HN, RNA-sequencing was performed on the kidneys of control mice and HN mice modeling for 14 days. Compared with control mice, the expression levels of 1367 genes were significantly altered in the kidneys of HN mice, of which 722 genes were upregulated and 655 genes were downregulated (Figure 2A, Table S1). Gene ontology (GO) analysis revealed that the differentially expressed genes (DEGs) were significantly enriched for genes involved in the regulation of oxidoreductase activity (*p* < 0.01) and oxidation-reduction process (*p* < 0.01) (Figure 2B,C). With further analysis, the up-regulated gene set was significantly enriched for genes involved in protein binding (*p* < 0.01), immune system process (*p* < 0.01), inflammatory response (*p* < 0.01), and apoptotic process (*p* < 0.01), many of which play critical roles in HN progression (Figure S2A,B) [24,25,26]; and the down-regulated gene set was significantly enriched for genes involved in oxidoreductase activity (*p* < 0.01) and the metabolic process (*p* < 0.01) (Figure S2C,D) [27,28].

Notably, HN associated with oxidative stress is incriminated in DNA damage, oxidations, inflammatory cytokine production, and even apoptosis [27]. Through the activation of genes encoding antioxidant and ROS-detoxifying enzymes, the NRF2 antioxidant pathway clears excess ROS and maintains redox homeostasis [19,29]. Remarkably, almost 40% of NRF2-targeted genes were changed in the kidney of HN mice, suggesting that the NRF2 signaling pathway may play an essential role in the progression of HN (Figure 2D). Of these, the transcription levels of 38 common antioxidant genes regulated by NRF2 were all down-regulated in the kidneys of HN mice, which play an essential protective role in scavenging ROS in kidney diseases, such as diabetic nephropathy, autosomal dominant polycystic kidney disease (ADPKD), and CKD (Figure 2E) [30,31,32]. In addition, the levels of antioxidant GSH and the activities of antioxidant enzymes GSH-Px and the activities of SOD were significantly down-regulated in the kidneys of HN mice, suggesting that the antioxidant signaling pathway was impaired (Figure 2F–H).

However, there was no significant change in the gene expression level of *nrf2* on the 14th day model in our RNA-sequencing data (Table S1); therefore, we detected changes of NRF2 expression and localization in different stages during HN progression. Interestingly, the expression and nuclear localization of NRF2 were increased early in disease progression (day 1 and day 3), with the highest nucleus level in day one. Subsequently, the expression of NRF2 gradually decreased as the HN progressed and finally fell below the baseline level (Figure 2I–K and Figure S2E). Collectively, the above results suggested that the NRF2 antioxidant signaling pathway was impaired in HN mice.

Considering the irreversible harm of HN conversion from AKI to CKD, we also characterized the NRF2 signaling pathway in the kidneys of CKD patients. We analyzed a microarray dataset containing microdissected renal tubules from the biopsies of healthy and CKD individuals. There was no significant difference in the expression of *nrf2* in the different kidney tissues of healthy people (Figure S2F). Compared with healthy people, *nrf2* was activated to varying degrees in the renal tubules of CKD patients, and the expression levels of *nrf2* were significantly increased in the kidneys of patients with diabetic nephropathy, IgG nephropathy, Lupus nephropathy, and microvascular disease (Figure S2G). In addition, the more severe the renal impairment indicated by Scr, BUN, and GFR, the higher the expression level of *nrf2* (Figure S2H–J). The above results indicate that the NRF2 signaling pathway plays an important role in the kidneys of both CKD patients and HN mice.

### 3.3. Loss of nrf2 Gene Aggravates Kidney Function Impairment in HN Mice

**Figure 3:**
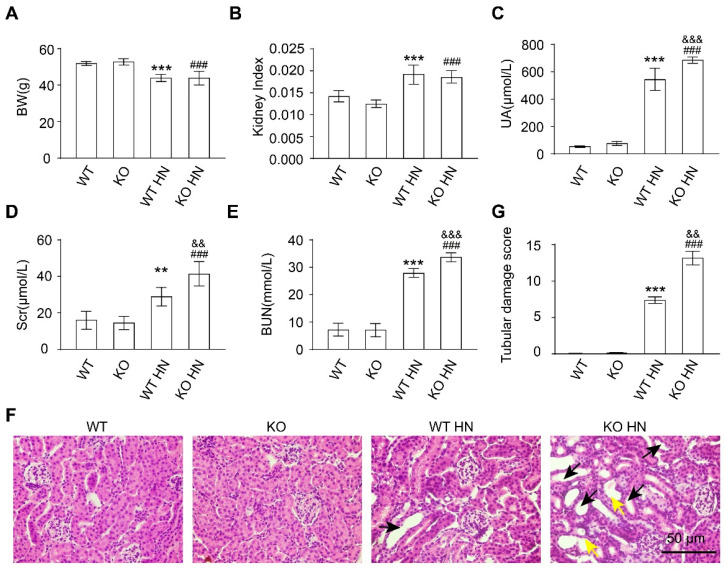
Deletion of the *nrf2* aggravates kidney damage in HN mice. (**A**) Body weight (BW) in the indicated groups. (**B**) Kidney index (kidney weight–to–body weight ratios) in the indicated groups. (**C**) UA in the indicated groups. (**D**) Scr in the indicated groups. (**E**) BUN in the indicated groups. (**F**) Hematoxylin and eosin (H&E) staining of kidney tissue. Black arrows represent dilation and yellow arrows represent loss of brush borders. Scale bar, 50 μm. (**G**) Quantification of tubular injury score in mouse kidney sections. Data represent means ± SEM. ** *p* < 0.01 and *** *p* < 0.001 vs. wild type (WT) mice. ^###^ *p* < 0.001 vs. *nrf2* knockout (KO) mice. ^&&^ *p* < 0.01 and ^&&&^ *p* < 0.001 vs. wild-type hyperuricemia nephropathy (WT HN) mice. Data are from the kidneys of six mice.

Subsequently, *nrf2* KO mice were used to confirm the critical role of NRF2 in HN development. Notably, KO mice died rapidly when induced with the original dose, i.e., 300 mg/kg hypoxanthine and 300 mg/kg potassium oxonate (Figure S3A). Therefore, according to the survival rate of KO HN mice under different induction doses and induction time conditions, we finally decided to use the induction doses of 150 mg/kg hypoxanthine and 300 mg/kg potassium oxonate via intraperitoneal injection for three consecutive days (Figure S3A,B). Wild type (WT) mice and KO mice have comparable body weight, kidney weight-to-body weight ratio, SUA, Scr, and BUN, indicating that the simple deletion of *nrf2* has little effect on the growth and renal function of mice (Figure 3A–E). However, the levels of SUA, Scr, and BUN in KO HN mice were significantly higher than HN mice, suggesting more severe renal function impairment in HN mice without *nrf2* (Figure 3A–E). In addition, H&E staining showed severe histopathological abnormalities in KO HN group, including tubular dilation and brush border loss (Figure 3F,G). These results indicated that the knockout of *nrf2* aggravated the renal injury, suggesting that *nrf2* plays an important role in maintaining kidney function and structure and HN development.

### 3.4. Activation of NRF2 Improves Kidney Function in HN Mice

Based on the above results, we hypothesized that activating NRF2 could delay the progress of HN. SFN is a Kelch-like ECH Associated Protein 1 (KEAP1) inhibitor that disrupts the KEAP1-NRF2 complex, thereby enhancing NRF2 expression and facilitating the translocation of NRF2 in the nucleus to regulate the expression of many antioxidants [33,34]. SFN has been used in basic research and clinical studies for many kidney diseases to reduce oxidative stress and inflammation [35,36,37]. Therefore, mice were injected with 300 mg/kg hypoxanthine and 300 mg/kg potassium oxonate mixture intraperitoneally for 14 consecutive days and given SFN (subcutaneous injection, 0.5 mg/kg and 2.5 mg/kg) for another 14 days to clarify the effects of NRF2 activation on renal injury in HN animal models (Figure S3C).

**Figure 4:**
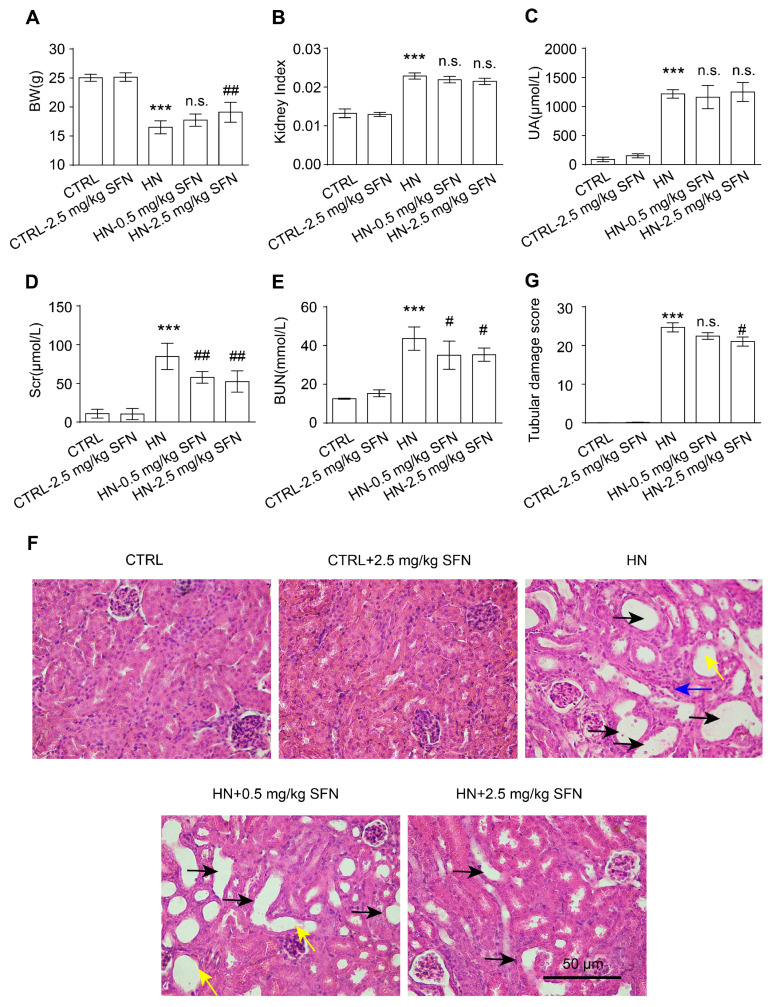
Activation of NRF2 improves kidney injury in HN mice. (**A**) Body weight (BW) in the indicated groups. (**B**) Kidney index (kidney weight–to–body weight ratios) in the indicated groups. (**C**) UA in the indicated groups. (**D**) Scr in the indicated groups. (**E**) BUN in the indicated groups. (**F**) Hematoxylin and eosin staining of kidney tissue. Black arrows represent dilation, blue arrows represent atrophy, and yellow arrows represent loss of brush borders. Scale bar, 50 μm. (**G**) Quantification of tubular injury score in mouse kidney sections. Data represent means ± SEM. *** *p* < 0.001 vs. control (CTRL) mice. ^#^ *p* < 0.05 and ^##^ *p* < 0.01 vs. hyperuricemia nephropathy (HN) mice. n.s. = no significance. Data are from the kidneys of six mice.

Treatment with high-dose SFN increased the body weight of HN mice, whereas neither low-dose nor high-dose SFN treatment had significant effects on kidney index or SUA levels in HN mice (Figure 4A–C). Notably, both the low-dose and high-dose SFN treatment significantly decreased Scr and BUN levels in HN mice, suggesting improved kidney function (Figure 4D,E). The histopathological staining results suggested that SFN treatment improved the renal structural damage in a dose-related manner in HN mice, including the restoration of glomerular morphology, the reduction in tubular dilatation and atrophy, the reduction in tubular brush border loss, and the reduction in tubular injury score (Figure 4F,G). There were no changes to the renal function and structure in normal mice given SFN (Figure 4C–G). Together, these results indicate that the activation of *nrf2* improves renal injury in HN mice.

### 3.5. Activation of NRF2 Improves Mitochondrial Dysfunction in Animal and Cell Models

**Figure 5:**
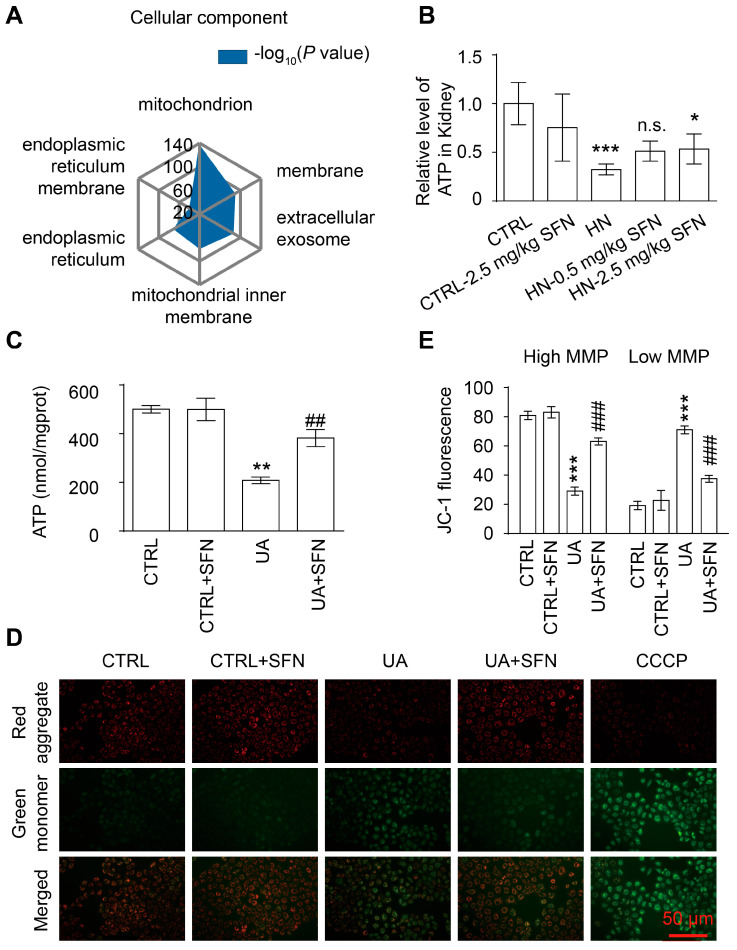
Activation of NRF2 ameliorates renal and cellular mitochondrial dysfunction in animal and cell models. (**A**) Gene ontology (GO) enrichment analysis of downregulated genes. (**B**) ATP levels in the kidneys in the indicated groups. (**C**) ATP levels of cells in cells in the indicated groups. (**D**) Representative images of MMP with the JC-1 staining of cells in the indicated groups, observed under a fluorescence microscope at 20× magnification. Carbonyl cyanide m-chlorophenyl hydrazine (CCCP) as positive control. Scale bar, 50 μm. (**E**) The ratio of JC-1 green and red fluorescence. Data represent means ± SEM. * *p* < 0.05, ** *p* < 0.01 and *** *p* < 0.001 vs. control (CTRL) group. ^##^ *p* < 0.01, and ^###^ *p* < 0.001 vs. hyperuricemia nephropathy (HN) group and uric acid (UA) group. n.s. = no significance. Data are from the kidneys of six mice, or data are from six replicate experiments.

We further investigated the molecular mechanism by which the activation of NRF2 ameliorates kidney injury in HN mice. GO analysis revealed that the downregulated oxidoreductase activity-related genes were related to mitochondria, one of the main sources of ROS in cells (Figure 5A). Based on this, we examined mitochondrial function in HN mice. ATP levels in the renal cortex of HN mice were significantly reduced compared to control mice but increased after a high dose of SFN treatment (Figure 5B). Renal tubular epithelial cells are one of the high energy-consuming cells, mainly relying on mitochondria for energy. To further elucidate the effect of SFN on mitochondria function in renal tubular epithelial cells, NRK-52E cells were stimulated with 8 mg/dL UA for 24 h in vitro, which resulted in decreased cellular ATP, while 0.5 μmol/L SFN treatment restored ATP (Figure 5C). Mitochondrial membrane potential (MMP) was detected using JC-1 dye. UA stimulation caused a decrease in MMPs in NRK-52E cells, which was restored through SFN treatment (Figure 5D,E).

### 3.6. Activation of NRF2 Rescues Mitochondrial Homeostasis in Animal and Cell Models

**Figure 6:**
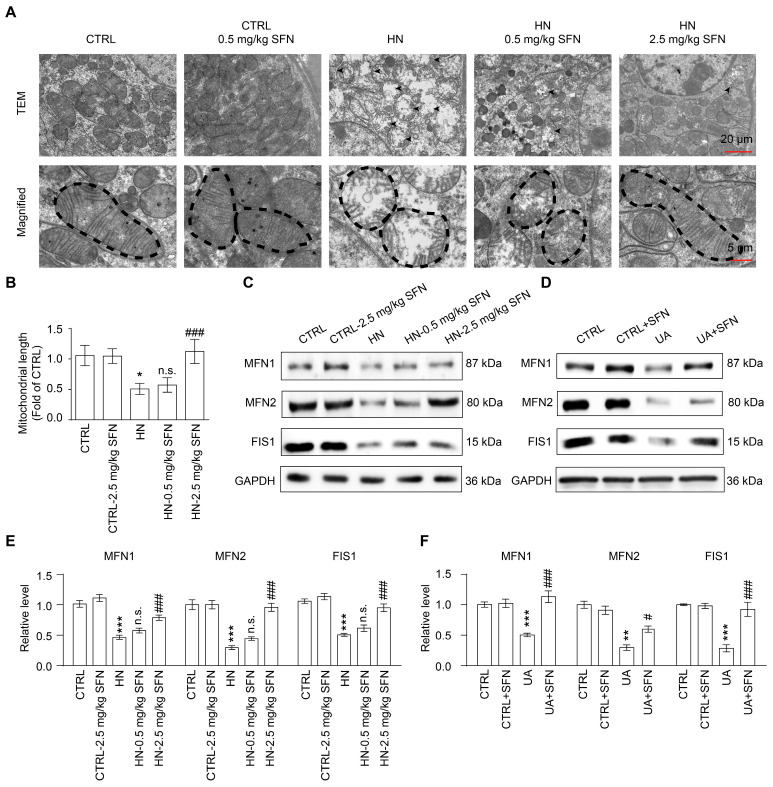
Activation of NRF2 restores renal and cellular mitochondrial homeostasis in animal and cell models. (**A**) Representative TEM images of mitochondria in the proximal tubular epithelial cells of HN mice, scale bar, 20 µm. (**B**) Quantification of the mitochondrial length detected by TEM. (**C**) Western blots of MFN1, MFN2, and FIS1 in the kidneys in the indicated groups, and the quantitative analysis of MFN1, MFN2, and FIS1 expression. (**D**) Western blots of MFN1, MFN2, and FIS1 in cells in the indicated groups. (**E**) The quantitative analysis of MFN1, MFN2, and FIS1 expression in (**C**). (**F**) The quantitative analysis of MFN1, MFN2, and FIS1 expression in (**D**). Data represent means ± SEM. * *p* < 0.05, ** *p* < 0.01 and *** *p* < 0.001 vs. control (CTRL) group. ^#^ *p* < 0.05 and ^###^ *p* < 0.001 vs. hyperuricemia nephropathy (HN) group and uric acid (UA) group. n.s. = no significance. Data are from kidneys of six mice, or data are from six replicate experiments.

To further analyze the molecular mechanism of mitochondrial dysfunction, cell mitochondrial ultrastructure in kidneys of mice was observed using an electron microscope. In vivo, proximal tubular epithelial cells from HN mice exhibited marked changes in mitochondrial morphology, including shortened mitochondrial length, mitochondrial swelling, loss of mitochondrial cristae, and reduced mitochondrial contacts with the endoplasmic reticulum, suggesting impaired mitochondrial function in HN kidneys (Figure 6A,B).

The maintenance of mitochondrial function and shape depends on mitochondrial fusion and fission processes, known as mitochondrial dynamics [38]. Mitofusin 1 (MFN1) and mitofusin 2 (MFN2) are mitochondrial fusion-related proteins, and mitochondrial fission protein 1 (FIS1) is a mitochondrial fission-related protein [38,39]. The results showed that the expression levels of MFN1, MFN2, and FIS1 were both downregulated in the renal cortex of HN mice and UA-stimulated cells. After SFN treatment, the expression levels of MFN1, MFN2, and FIS1 were upregulated, suggesting that the activation of NRF2 can restore the ability of mitochondrial fusion and fission to improve mitochondrial dysfunction (Figure 6C–F).

### 3.7. Activation of NRF2 Alleviates Oxidative Stress in Animal and Cell Models

**Figure 7:**
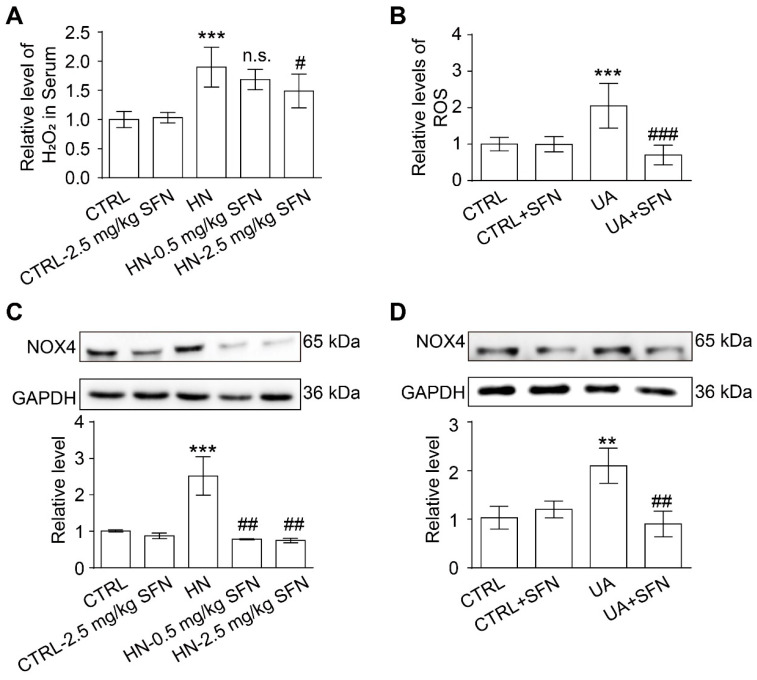
Activation of NRF2 relieves kidney and cellular oxidative stress in animal and cell models. (**A**) The level of H_2_O_2_ in the serum of mice in the indicated groups. (**B**) Quantitative analysis of ROS levels in cells in the indicated groups. (**C**) Western blot of NOX4 in the kidneys of mice in the indicated groups, and the quantitative analysis of NOX4 expression. (**D**) Western blot of NOX4 in cells in the indicated groups, and the quantitative analysis of NOX4 expression. Data represent means ± SEM. ** *p* < 0.01 and *** *p* < 0.001 vs. control (CTRL) group. ^#^ *p* < 0.05, ^##^ *p* < 0.01, and ^###^ *p* < 0.001 vs. hyperuricemia nephropathy (HN) group and uric acid (UA) group. n.s. = no significance. Data are from the kidneys of six mice, or data are from six replicate experiments.

Mitochondria and NOX4 are the main sources of ROS in the kidney; therefore, ROS levels and NOX4 expression were examined in HN animal model and UA-stimulated cell model. As one of the main components of ROS, there was a significant upregulation of H_2_O_2_ levels in serum in HN mice compared with control mice, and SFN treatment significantly reduced H_2_O_2_ levels (Figure 7A). In the cell model, UA stimulation increased the level of ROS in cells, while SFN treatment significantly reduced the generation of cellular ROS, which did not affect the level of ROS in normal cells (Figure 7B).

Western blot results showed that the expression of NOX4 in the renal cortex of HN mice was significantly higher than that of control mice, and SFN treatment significantly down-regulated the expression of NOX4 in the renal cortex of HN mice (Figure 7C). For cells, UA stimulation increased the expression of NOX4 and SFN treatment significantly reduced the expression of NOX4 (Figure 7D). The above results suggest that the activation of NRF2 may be alleviating oxidative stress by restoring mitochondrial dysfunction and down-regulating NOX4 expression.

### 3.8. Activation of NRF2 Enhances the Antioxidant Capacity of Animals and Cells by Up-Regulating NRF2/HO-1/NQO1 Signaling Pathway

**Figure 8:**
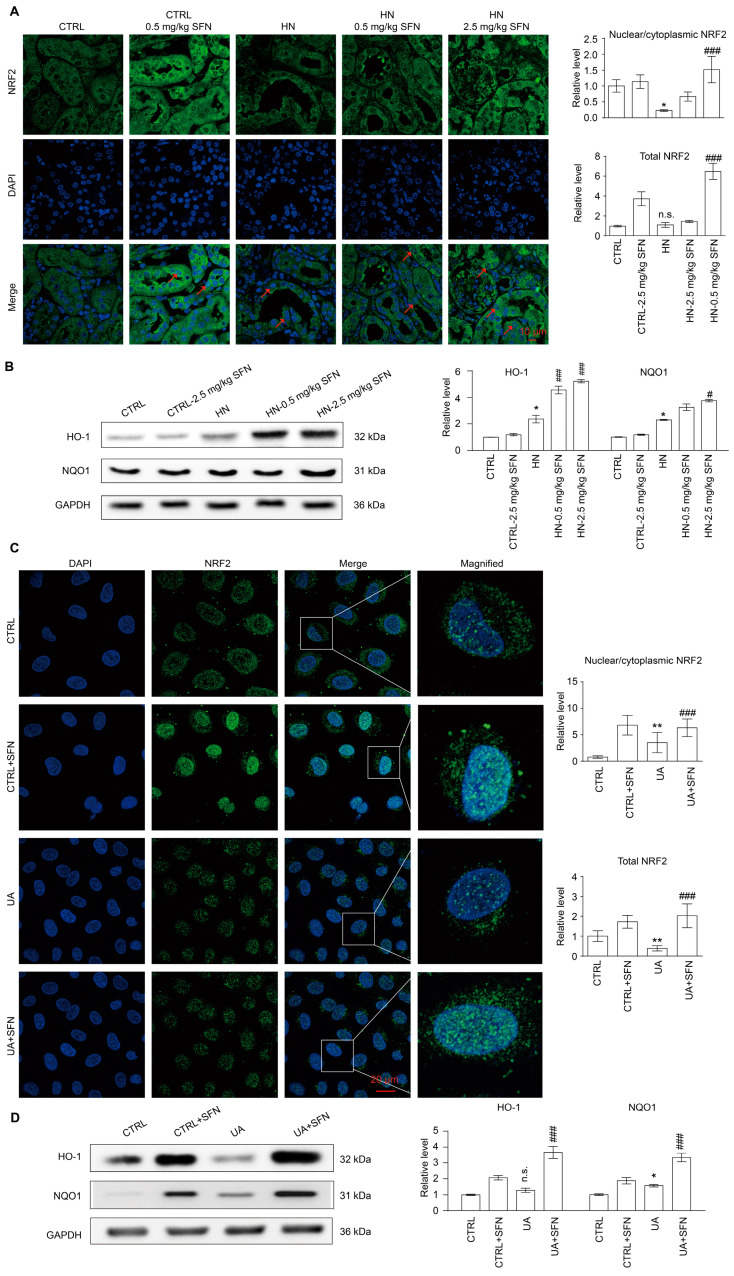
Activation of NRF2 enhances the antioxidant capacity of HN mouse kidneys and cells. (**A**) Immunofluorescence staining of NRF2 in kidneys in the indicated groups, the quantification of the expression of overall NRF2 in (**A**), and the quantification of the ratio of NRF2 in the nucleus to NRF2 in the cytoplasm in (**A**). Scale bar, 10 μm. (**B**) Western blots of HO-1 and NQO1 in the kidneys in the indicated groups, and the quantitative analysis of the expression of HO-1 and NQO1. (**C**) Immunofluorescence staining of NRF2 in cells in the indicated groups, the quantification of the expression of overall NRF2 in (**C**), and the quantification of the ratio of NRF2 in the nucleus to NRF2 in the cytoplasm in (**C**). The inset shows a higher magnification of the rectangular area. Scale bar, 20 μm. (**D**) Western blots of HO-1 and NQO1 in the cells in the indicated groups, and the quantitative analysis of the expression of HO-1 and NQO1. Red arrows indicate NRF2 nuclear localization. DAPI, 4’,6-diamidino-2-Phenyl indole. Data represent means ± SEM. * *p* < 0.05 and ** *p* < 0.01 vs. control (CTRL) group. ^#^ *p* < 0.05 and ^###^ *p* < 0.001 vs. hyperuricemia nephropathy (HN) group and uric acid (UA) group. n.s. = no significance. Data are from the kidneys of six mice, or data are from six replicate experiments.

Considering that oxidative stress usually occurs in the early stage of HN and the activity of NRF2 often undergoes dynamic changes during disease progression, we found that the ROS level in vitro was consistent with the changing trend of NRF2 expression and nuclear localization. Both increased rapidly in the early stage of UA stimulation, and then decreased (Figure S4). In addition, the expression and nuclear localization of NRF2 decreased in the kidneys of HN mice, while SFN administration was able to promote its expression and nuclear localization but had no effect on *nrf2^−/−^* mice, indicating that the NRF2 signaling pathway was activated in HN mice after SFN treatment (Figure 8A and Figure S5A).

The expression levels of key antioxidant enzymes heme oxygenase 1(HO-1) and quinone oxidoreductase 1 (NQO1) regulated by NRF2 were also detected. The results showed that the expression levels of HO-1 and NQO1 in the renal cortex of HN mice were significantly increased (Figure 8B). SFN treatment did not affect the expression levels of HO-1 and NQO1 in the renal cortex of control mice but further increased the expression levels of HO-1 and NQO1 in the renal cortex of HN mice (Figure 8B).

We observed the cellular localization of NRF2 in renal tubular epithelial cells. UA stimulation decreased the overall expression of NRF2 in cells and increased the proportion of nuclear localization. SFN treatment up-regulated the decrease in NRF2 expression induced by UA stimulation and further increased the nuclear localization ratio of NRF2 (Figure 8C). In the cell model, compared with the control group, UA stimulation caused an increase in the expression of HO-1 and NQO1 in the cells. The administration of SFN significantly increased the expression of HO-1 and NQO1 in the cells of the control group, and further increased the expression of HO-1 and NQO1 in the UA group (Figure 8D). The results suggested that the activation of NRF2 significantly improved the antioxidant capacity of the HN mouse kidney cortex and UA cell model.

### 3.9. Activation of NRF2 Improves Renal Fibrosis in Animal and Cell Models by Down-Regulating TGF-β1/α-SMA/Collagen 1 Signaling Pathway

**Figure 9:**
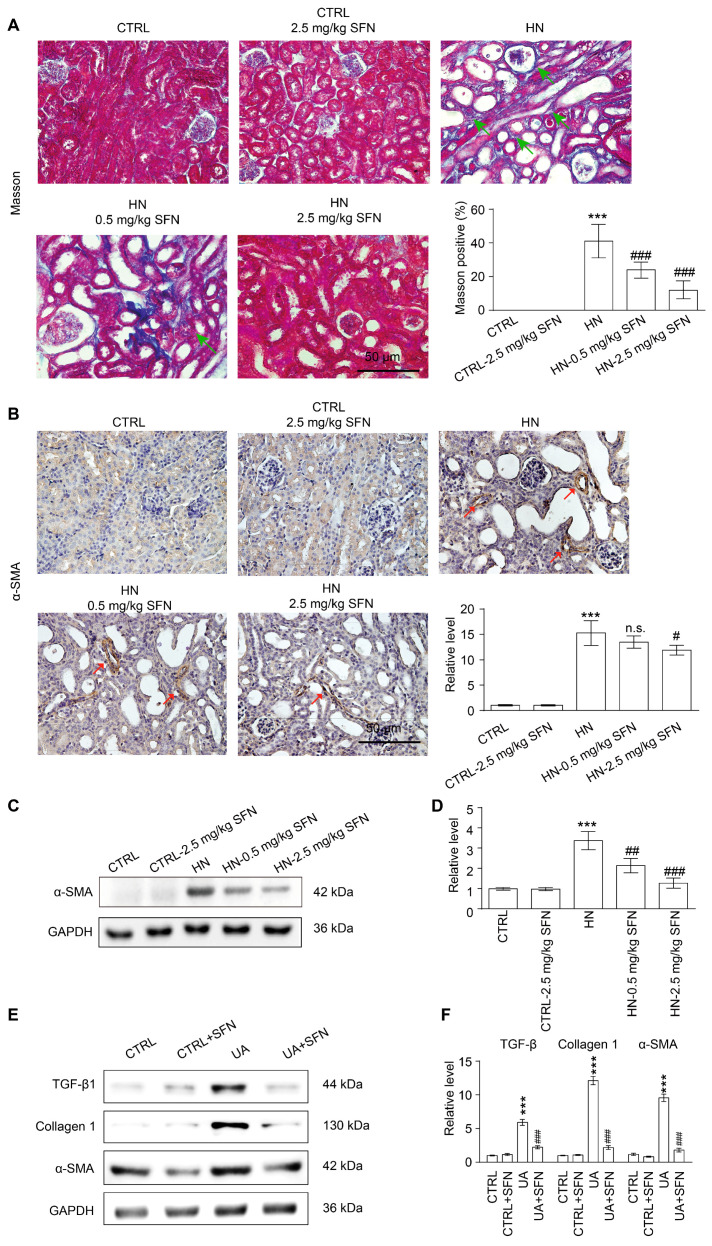
Activation of NRF2 ameliorates renal fibrosis in animal and cell models. (**A**) Masson staining of kidney tissue and the quantification of Masson-stained positive areas in mouse kidney sections, green arrows represent collagen deposits that are stained blue. Scale bar, 50 μm. (**B**) Immunohistochemical staining of α-SMA in kidneys in the indicated groups, red arrows represent α-SMA-positive areas. Scale bar, 50 μm. (**C**) Western blot of α-SMA in the kidneys in the indicated groups. (**D**) Quantitative analysis of α-SMA expression in (**C**). (**E**) Western blots of TGF-β1, collagen 1, and α-SMA in the indicated groups. (**F**) Quantitative analysis of the expression of TGF-β1, collagen 1, and α-SMA in (**E**). Data represent means ± SEM. *** *p* < 0.001 vs. control (CTRL) group. ^#^ *p* < 0.05, ^##^ *p* < 0.01 and ^###^ *p* < 0.001 vs. hyperuricemia nephropathy (HN) group and uric acid (UA) group. n.s. = no significance. Data are from the kidneys of six mice, or data are from six replicate experiments.

The deletion of *nrf2* did not affect collagen fiber deposition and α-SMA expression in mouse kidneys, but KO HN mice showed higher levels of fibrosis and α-SMA expression compared with WT HN mice (Figure S5B–D). As a common consequence of kidney disease, the kidneys of HN mice developed banded interstitial fibrosis accompanied by the proliferation of collagen fibers compared with control mice, whereas SFN treatment significantly reduced the level of fibrosis (Figure 9A). In the renal cortex of HN mice, the expression of α-SMA was increased, whereas SFN treatment dose-relatedly decreased the expression of α-SMA (Figure 9B–D). This suggests that NRF2 plays an important role in the progression of renal fibrosis.

We further explored the molecular mechanism by which the activation of NRF2 signaling improves renal fibrosis in renal tubular epithelial cells. TGF-β1 is a pro-fibrotic factor. In the NRK-52E cell model, UA stimulation up-regulated the expression of TGF-β1, α-SMA, and collagen 1, which could be reversed by SFN (Figure 9E,F). Taken together, the results indicated that SFN ameliorates renal fibrosis in HN mice by inhibiting the TGF-β1/α-SMA/collagen 1 signaling pathway.

## 4. Discussion

The mechanisms of HUA-induced renal injury mainly include oxidative stress, inflammation, endothelial dysfunction, and renal fibrosis. Although many studies have shown that decreased SUA levels are associated with renal protection, some studies indicated that XO inhibitors may exert renal protection beyond lowering UA, because XO is also a critical source of ROS and the inhibition of XO by allopurinol or febuxostat attenuates ROS-mediated kidney injury, which reflects the importance of antioxidant in HUA-induced renal injury [40,41].

### 4.1. NRF2 Antioxidant Signaling Pathway Is Impaired in the Kidney of HN Mice

To elucidate the key signaling pathways of HUA-induced renal injury, we performed the RNA-sequencing of the renal cortex from HN mice and normal mice. The results showed that gene-related oxidoreductase activity and the oxidation-reduction process played an important role in our HN model, while the relationship between oxidative stress and HN was unclear. In the present study, elevated SUA levels were significantly and positively correlated with Scr and BUN, while GFR was negatively correlated. These results from HN mouse model suggest a progressive decline in kidney function with elevated SUA levels. Meanwhile, some anti-oxidative molecules, such as GSH, GSH-Px, and SOD, decreased in the cortex of kidneys of HN mice as the consistent results of RNA-sequencing (Figure 2). Thus, we hypothesize that sustained high-SUA levels can trigger renal oxidative stress which might contribute to HUA-induced renal injury.

The microarray dataset gave us the clue that NRF2-activated signaling pathway contributes to the development of many kidney diseases. Interestingly, with the results of immunofluorescence and immunohistochemical staining, NRF2 changed in a different small way, which was slightly activated in the early HN model (day 1 and day 3) and then decreased in the developing and late HN model (day 5, 7, and 14). As the indicator and regulator of oxidative stress, NRF2-antioxidant response element (ARE) pathway has been shown to have dynamic changes and play a renoprotective role in many kidney diseases, but its role in HN is unknown. Under oxidative stress conditions, NRF2 enters the nucleus to regulate the expression of antioxidant or detoxification genes [19]. Many studies have already shown that the NRF2 signaling pathway is often activated in the early stage of the disease to play a positive role against oxidation, which was consistent with the up-regulation of NRF2 expression and nuclear translocation in our study. With the further increase in SUA level, the renal function and the NRF2 signaling pathway of HUA mice were impaired. Combined with the results of H&E and Masson staining, the HN model further exhibited multifarious features and pathological conditions with obvious fibrosis, suggesting the transition from AKI to CKD in HN mice. Therefore, we speculate that the antioxidant signaling pathway activated by NRF2 has an important protective effect on the progression of HN, especially in the early stages of renal injury, and put forward the scientific hypothesis that the activation of the NRF2 signaling pathway can delay the progression of HN.

### 4.2. Activation of NRF2 Improves Kidney Function in HN Mice

The scientific hypothesis above was tested and verified by pharmacologically activating NRF2 signaling and knocking out the *nrf2* gene. Firstly, to further identify the role of NRF2 in HN development, KO mice were injected with potassium oxonate (300 mg/kg) and hypoxanthine (150 mg/kg) for 3 days to establish an HN mouse model with *nrf2* deletion. Because all the animals would die under the original modeling conditions in WT mice, we reduced the dose and time of induction. Without HN induction, the deletion of *nrf2* in mice showed similar renal function as the wild-type mice. However, HN mice with *nrf2* deletion showed more severe kidney dysfunction and fibrosis pathological features, which may indirectly lead to a further decrease in UA excretion and an increase in blood UA levels. Therefore, the knockout of the *nrf2* gene significantly aggravated renal dysfunction and accelerated disease progression in HN mice, indicating a “vicious cycle” between SUA levels and renal dysfunction. As a result, the activation of NRF2 may be an effective strategy to decrease oxidative stress and renal fibrosis, as well as improve kidney function to ultimately delay HN progression.

SFN, a natural compound derived from cruciferous vegetables, is a relatively well-recognized KEAP1 inhibitor that disrupts the KEAP1–NRF2 complex, thereby promoting the nuclear accumulation of NRF2. Our study showed that SFN treatment significantly improved renal function and histopathological damage in HN mice without a urate-lowering effect.

### 4.3. Activation of NRF2 Improves Mitochondrial Dysfunction by Improving Mitochondrial Homeostasis in Animal and Cell Models

Subsequently, we further explored the molecular mechanism by which the activation of the NRF2 signaling pathway alleviates the progression of HN. GO analysis indicated that the downregulated redox signaling pathway was mainly localized in the mitochondria of cells. Mitochondria, dynamic organelles, generate ATP to provide an energy source for the kidney cell, which is critical for maintaining the normal function of tubular cell [42,43]. On the opposite, mitochondrial dysfunction increases the risks of tubulointerstitial disease, cystic kidney disease, podocytosis, and nephrotic syndrome [42]. We found that the ability of mitochondrial ATP production in HN mice was decreased, the mitochondrial structure was changed, and the mitochondrial function was impaired. Furthermore, the causes of abnormal mitochondrial structure and dysfunction were investigated.

Mitochondria can autonomously translate cytosol signals into directional regulatory signals for mitochondrial physiology and maintain normal conditions through the processes of mitochondrial fusion and fission. Fusion and fission are processes that create homeostasis, known as the mitochondrial dynamics, which are essential for healthy stem cell development, self-renewal and differentiation, and overall cellular fitness [44,45,46]. Imbalances of mitochondrial dynamics contribute to the progression of neurodegenerative diseases, cancer, heart disease, and other diseases [47]. Our results showed the altered mitochondrial ultrastructure and mitochondrial dysfunction in the kidneys of HN mice, attributed to the impaired ability of mitochondria to fuse and fission, that is, an imbalance in mitochondrial dynamics.

In addition, mitochondria have the ability to generate ATP and provide energy for life activities by pumping protons (H^+^) into the mitochondrial membrane. If damaged, the membrane permeability of mitochondria increases, resulting in the free movement of H+ through the mitochondrial membrane [48]. Subsequently, reduced MMP leads to irreversible apoptosis [49]. Without enough ATP, calcium pumps cannot function properly, and calcium overloads. Eventually, the mitochondria swell and the mitochondrial structure is disrupted. It has been revealed that decreased MMP occurs in certain renal diseases, such as Fanconi syndrome, Leigh disease, and focal segmental glomerulosclerosis [50,51,52,53]. Our study showed that high levels of UA lead to ATP depletion, mitochondrial MMP reduction, mitochondrial structural disruption, and mitochondrial edema in renal tubular epithelial cells, while SFN treatment reduced the mitochondrial damage and restored the ability of mitochondrial fusion and fission in the kidneys of HN mice. Therefore, the activation of the NRF2 signaling pathway is able to improve mitochondrial dysfunction by restoring mitochondrial MMPs and mitochondrial dynamic imbalance.

### 4.4. Activation of NRF2 Alleviates Oxidative Stress in Animal and Cell Models

Mitochondria is one of the main sources of ROS in cells [54]. In our study, high levels of UA lead to ROS overproduction and cellular oxidative stress in the epithelioid cell line of a normal rat kidney (NRK-52E cell), while SFN activates NRF2 signaling to inhibit ROS production and ameliorate mitochondrial dysfunction. In addition to mitochondria, NOX4 is another major source of ROS in the kidney, including H_2_O_2_ and superoxide anion [54,55]. Some studies have already shown that UA causes the activation of NOX, which furthers transfers to mitochondria and increases mitochondrial ROS production [56,57,58]. Our results demonstrate that HN mice developed systemic and renal oxidative stress, manifested by increased serum H_2_O_2_ levels and upregulated renal NOX4 expression, which were reversed through the activation of NRF2 signaling. All these suggest that the activation of NRF2 signaling is able to reduce mitochondria and NOX4-derived ROS, thereby alleviating oxidative stress in the kidneys of HN mice and improving renal function.

### 4.5. Activation of NRF2 Enhances the Antioxidant Capacity of Animals and Cells by Up-Regulating NRF2/HO-1/NQO1 Signaling Pathway

To resist oxidative stress, NRF2 will translocate to the nucleus to regulate the expression of certain antioxidant proteins or enzymes and phase II detoxification enzyme genes. Of these, NQO1 and HO-1 are the key target genes regulated by NRF2. Consistent with the reported results, although the expressions of NOQ1 and HO-1 were upregulated in the renal cortex and tubular epithelial cells stimulated by high levels of UA, the changes were not able to completely prevent the occurrence of oxidative stress [59]. The activation of the NRF2 signaling pathway by SFN can further up-regulate the expression of NQO1 and HO-1 in kidney and renal tubular epithelial cells, enhance the ability of cells to resist oxidative stress, and help relieve oxidative stress.

### 4.6. Activation of NRF2 Improves Renal Fibrosis in Animal and Cell Models by Down-Regulating TGF-β1/α-SMA/Collagen 1 Signaling Pathway

Besides the mitochondrial dysfunction and oxidative stress, the long-term high levels of SUA also caused renal interstitial fibrosis, similarly to the final common pathway and histological manifestation of CKD. As clarified in other studies, excess oxidative stress induced renal interstitial fibrosis in many pathological conditions [60,61], which was also shown in this study. High levels of UA caused the increased expression of fibrosis markers (TGF-β1, α-SMA, and collagen 1) both in the HN mice and the NRK-52E cells, suggesting the activation of the pro-fibrotic signaling pathway. SFN treatment down-regulated the expression levels of these fibrosis markers, indicating that the activation of the NRF2 signaling pathway alleviated the renal fibrosis and delayed the progression from AKI to CKD in HN mice.

**Figure 10:**
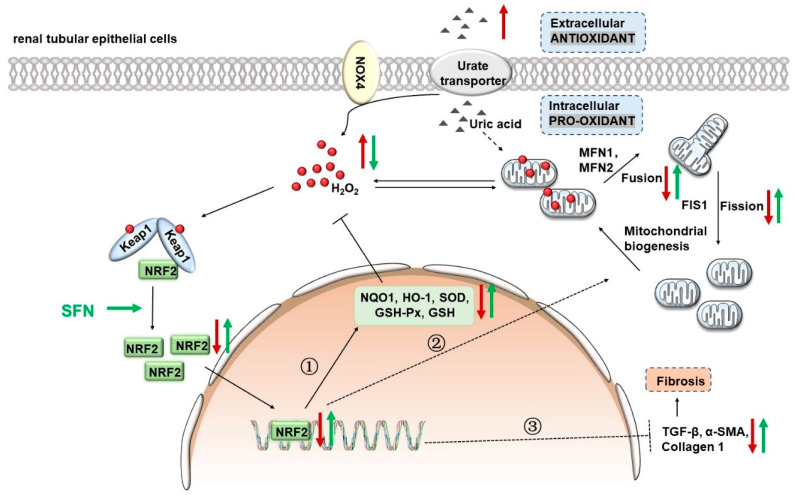
Mechanisms by which the activation of NRF2 signaling ameliorates kidney injury in HN mice. Under physiological conditions, cells produce an appropriate amount of ROS to adapt to normal physiological activities, while NRF2 exists in the cytoplasm at a lower level. High levels of UA will be transported into cells by transporters on the apical membrane of renal tubular epithelial cells, stimulating NOX4 to produce excessive ROS. Excessive ROS causes mitochondrial fusion and fission disorders, leading to mitochondrial dysfunction. Damaged mitochondria produce excessive mitochondrial ROS and aggravate cellular oxidative stress. The administration of NRF2 agonist sulforaphane (SFN) can improve cellular oxidative stress by down-regulating NOX4 expression and remodeling mitochondrial homeostasis. Moreover, NRF2 activation enhances the antioxidant capacity of cells by upregulating the expression of HO-1/NQO1, reduces renal fibrosis by downregulating the expression of TGF-β1/α-SMA/Collagen 1, and ultimately improves renal function and slows down disease progression.

In summary, this study focuses on the role and specific molecular mechanism of NRF2 in the development of HN. The activation of NRF2 signaling improved renal function damage and delayed disease progression in HN mice by restoring the mitochondrial homeostasis and reducing the oxidative stress. Moreover, NRF2 activation enhances the antioxidant capacity of cells by upregulating the expression of HO-1/NQO1, reduces renal fibrosis by downregulating the expression of TGF-β1-dependent profibrotic signals, and ultimately improves renal function and slows down HN progression (Figure 10). At present, clinical trials on the effect of NRF2 agonists on diabetic nephropathy, CKD, or other kidney diseases have been completed or are underway (NCT03550443, NCT03019185, NCT03749447, NCT03366337, and NCT03918447). This study suggests that NRF2 agonists are promising for CKD caused by HN. Our findings for the first time reveal NRF2 to be a validated therapeutic target against HN, and chemicals targeting NRF2 might be promising agents in treating HN-related AKI and CKD.

## 5. Conclusions

This study found for the first time that the antioxidant signaling pathway activated by NRF2 plays an important role in the occurrence and development of HN. The activation of the NRF2-regulated antioxidant signaling pathway, the down-regulation of NOX4 expression, and the improvement of mitochondrial dysfunction are able to alleviate renal oxidative stress injury. Furthermore, by down-regulating the TGF-β1-dependent pro-fibrotic signaling pathway in HN mice, tubulointerstitial fibrosis is inhibited and the transition from AKI to CKD is delayed. The results of this study suggest that NRF2 may be a potential target for delaying the progression of HN, and activating the NRF2 signaling pathway may be an effective strategy for improving HN or delaying the progression of CKD.

## Data Availability

All data produced in this study are provided in the manuscript and Supplementary Materials.

## Supplementary Materials

The following supporting information can be downloaded at: https://www.mdpi.com/article/10.3390/antiox12051022/s1. Figure S1: (A) Schematic diagram of induction patterns of hyperuricemia (HUA) mouse models with different progressions. PO = potassium oxonate, HX = hypoxanthine. (B) Urine output of mice in indicated groups. (C) Uric acid (UA) levels in the urine of the indicated groups of mice. (D) The osmolarity of the urine of the indicated groups of mice. Data represent means ± SEM. *** *p* < 0.001 vs. wild-type mice injected with inducer for 0 day. Data are from kidneys from six mice. Figure S2: (A and B) Gene ontology (GO) enrichment analysis of downregulated genes. (C and D) Gene ontology (GO) enrichment analysis of upregulated genes. Data are from kidneys from three mice. © Immunohistochemical staining of NRF2 in kidneys in the indicated groups. Scale bar, 20 μm. Arrows indicate NRF2 nuclear localization. DAPI, 4’,6-diamidino-2-Phenyl indole. The inset shows a higher magnification of the rectangular area. (F) Microarray analysis showing Tukey’s box plots of the site-specific, expression of NRF2 in different kidney tissues of healthy individuals (*n* = 34). (G) Expression of NRF2 in renal tubular samples from healthy individuals and CKD patients (*n* = 201). (H to J) The relationship between the expression of *nrf2* gene and serum creatinine (Scr), blood urea nitrogen (BUN) and glomerular filtration rate (GFR). * *p* < 0.05, ** *p* < 0.01, *** *p* < 0.001 vs. healthy living donor group, less than 1 mg/dL Scr group, less than 20 mg/dL group, and less than 15 mg/dL group. Figure S3: (A) Survival curve of hyperuricemia nephropathy (HN) model induced by *nrf2* knockout mice. Data are from the kidneys from 25 mice. (B) Schematic diagram of the induction model of HN induced by *nrf2* knockout mice. (C) Schematic diagram of the induced model of HN mouse model treated with SFN. SFN = sulforaphane, PO = potassium oxonate, HX = hypoxanthine. Figure S4: The production level of ROS after uric acid stimulated NRK-52E cells for 0, 0.5, 1, 2, 4, and 24 h, respectively (up), the expression and distribution of NRF2 after uric acid stimulated NRK-52E cells for 0, 0.5, 1, 2, 4, and 24 h, respectively (down). Scale bar, 50 μm. Data are from six replicate experiments. Figure S5: (A) Immunofluorescence staining of NRF2 in kidneys in the indicated groups. Scale bar, 10 μm. (B) Masson staining of kidney tissue. Green arrows represent collagen deposits that are stained blue. Scale bar, 50 μm. (C) Western blot of α-SMA in the kidneys in the indicated groups. (D) Quantitative analysis of the expression of α-SMA. Data represent means ± SEM. * *p* < 0.05 vs. wild type (WT) group. ^###^ *p* < 0.001 vs. *nrf2* knockout (KO) group, ^&&&^ *p* < 0.001 vs. wild-type hyperuricemia nephropathy (WT HN) group. Data are from the kidneys of four mice. Table S1: Information on differentially expressed genes.
