# Supplementary material for: Activation of NRF2 Signaling Pathway Delays the Progression of Hyperuricemic Nephropathy by Reducing Oxidative Stress
Source: Antioxidants (Basel). 2023 Apr 28;12(5):1022. doi: 10.3390/antiox12051022 (PMC10215166; doi:10.3390/antiox12051022)
Supplement: Supplementary file 1 [file antioxidants-12-01022-s001.zip › Supplementary_Material1.pdf]

Supplementary Material

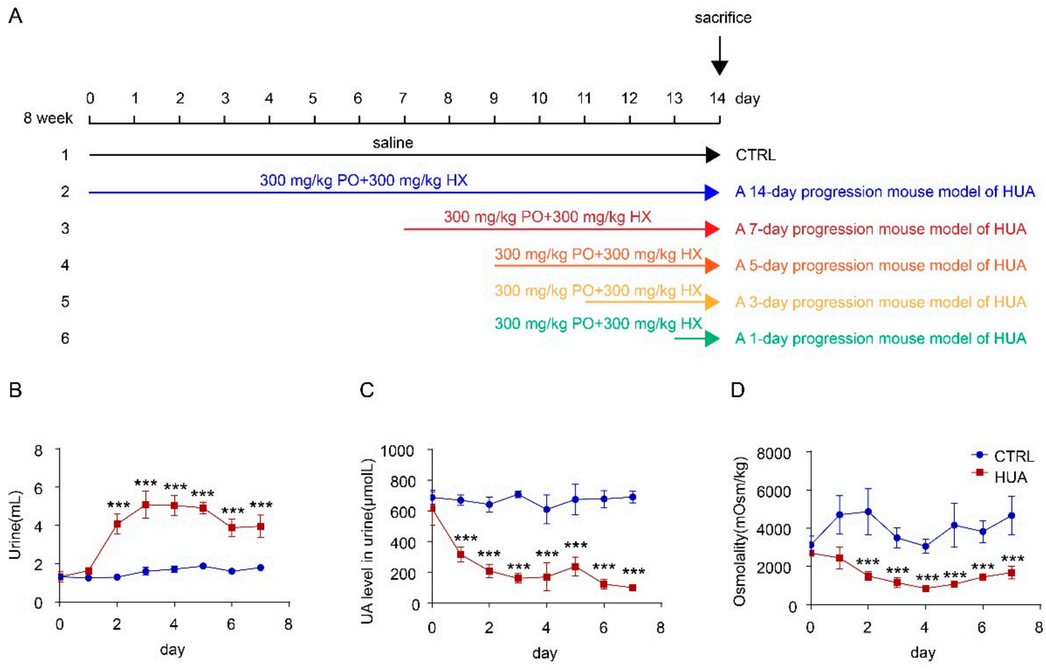

Figure S1 | (A) Schematic diagram of induction patterns of hyperuricemia (HUA) mouse models with different progressions. PO=potassium oxonate, HX=hypoxanthine. (B) Urine output of mice in indicated groups. (C) Uric acid (UA) levels in the urine of the indicated groups of mice. (D) The osmolarity of the urine of the indicated groups of mice. Data represent means ± SEM. ***$P < 0.001$ *vs.* wild-type mice injected with inducer for 0 day. Data are from kidneys from six mice.

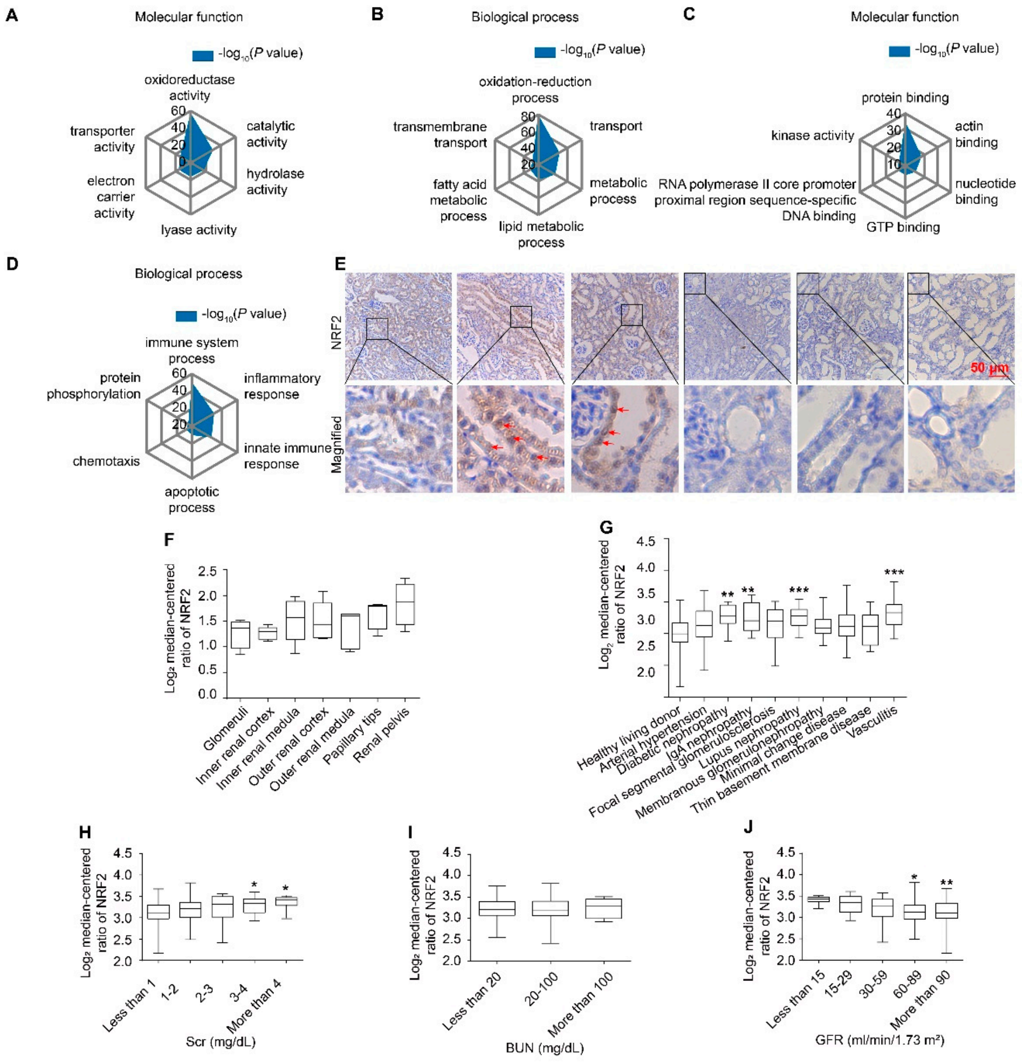

Figure S2 | (A and B) Gene Ontology (GO) enrichment analysis of downregulated genes. (C and D) Gene Ontology (GO) enrichment analysis of upregulated genes. Data are from kidneys from three mice. (E) Immunohistochemical staining of NRF2 in kidneys in the indicated groups. Scale bar, 20 μm. Arrows indicate NRF2 nuclear localization. DAPI, 4',6-diamidino-2-Phenyl indole. The inset shows a higher magnification of the rectangular area. (F) Microarray analysis showing Tukey box plots of site-specific, expression of NRF2 in different kidney tissues of healthy individuals (n=34). (G) Expression of NRF2 in renal tubular samples from healthy individuals and

CKD patients (n=201). (H to J) The relationship between the expression of *nrf2* gene and serum creatinine (Scr), blood urea nitrogen (BUN) and glomerular filtration rate (GFR). *$P < 0.05$, **$P < 0.01$, ***$P < 0.001$ *vs.* healthy living donor group, less than 1 mg/dL Scr group, less than 20 mg/dL group, and less than 15 mg/dL group.

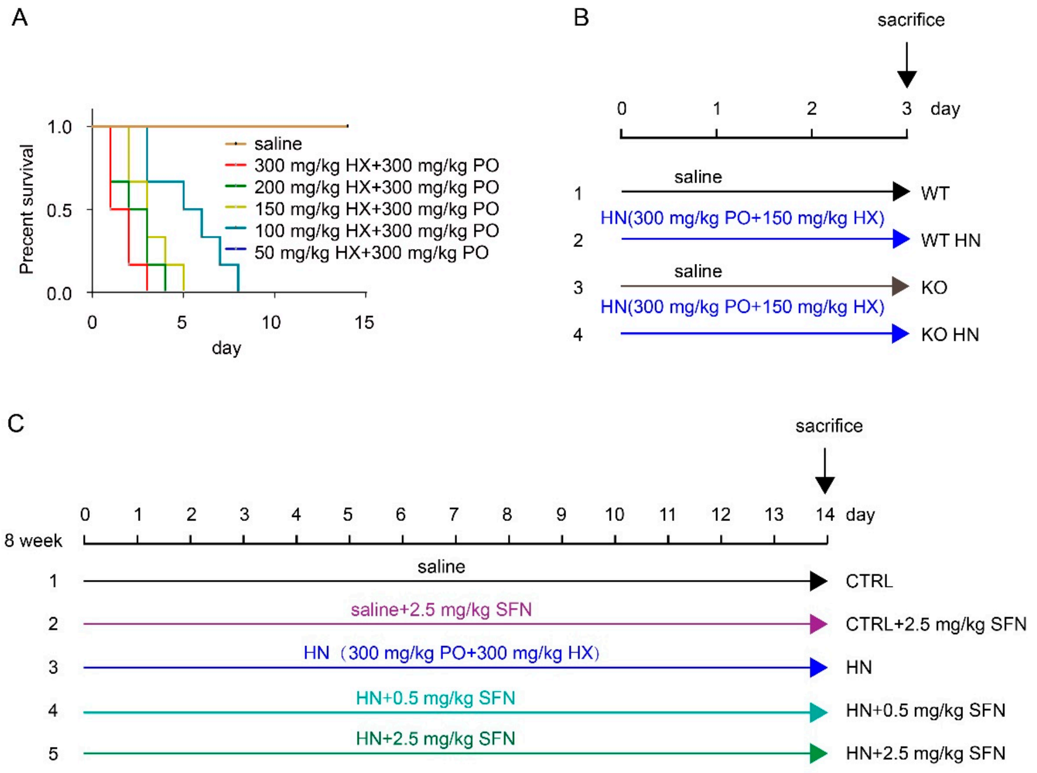

Figure S3 | (A) Survival curve of hyperuricemia nephropathy (HN) model induced by *nrf2* knockout mice. Data are from kidneys from 25 mice. (B) Schematic diagram of the induction model of HN induced by *nrf2* knockout mice. (C) Schematic diagram of the induced model of HN mouse model treated with SFN. SFN=sulforaphane, PO=potassium oxonate, HX=hypoxanthine.

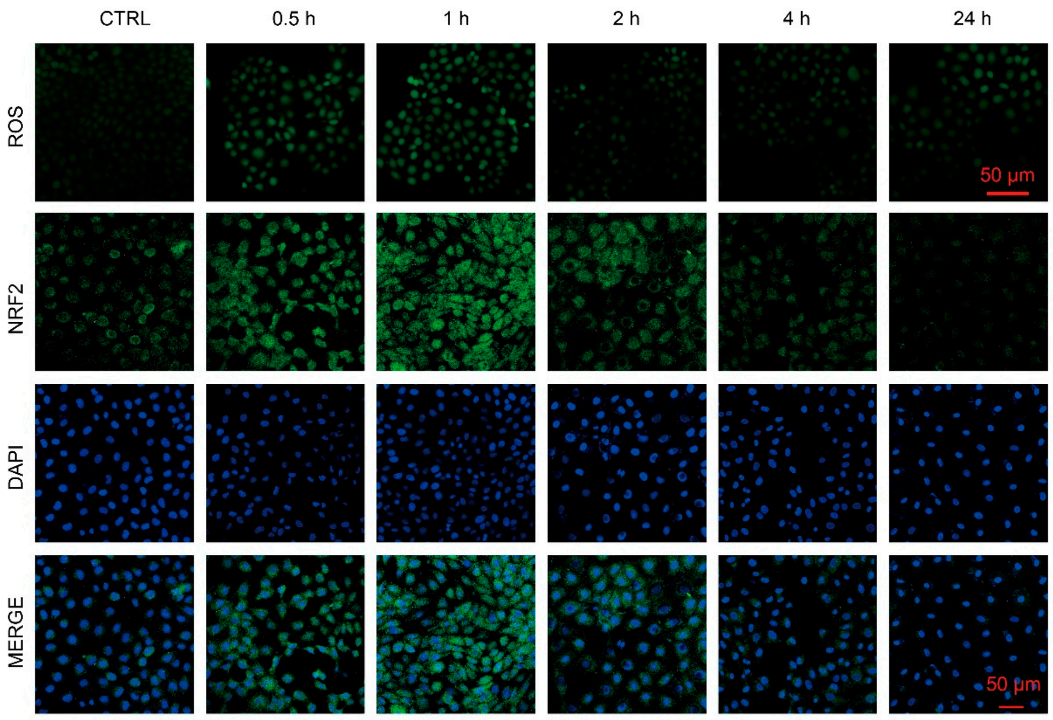

Figure S4 | The production level of ROS after uric acid stimulated NRK-52E cells for 0, 0.5, 1, 2, 4, and 24 hours respectively (up), the expression and distribution of NRF2 after uric acid stimulated NRK-52E cells for 0, 0.5, 1, 2, 4 and 24 hours respectively (down). Scale bar, 50 μm. Data are from six replicate experiments.

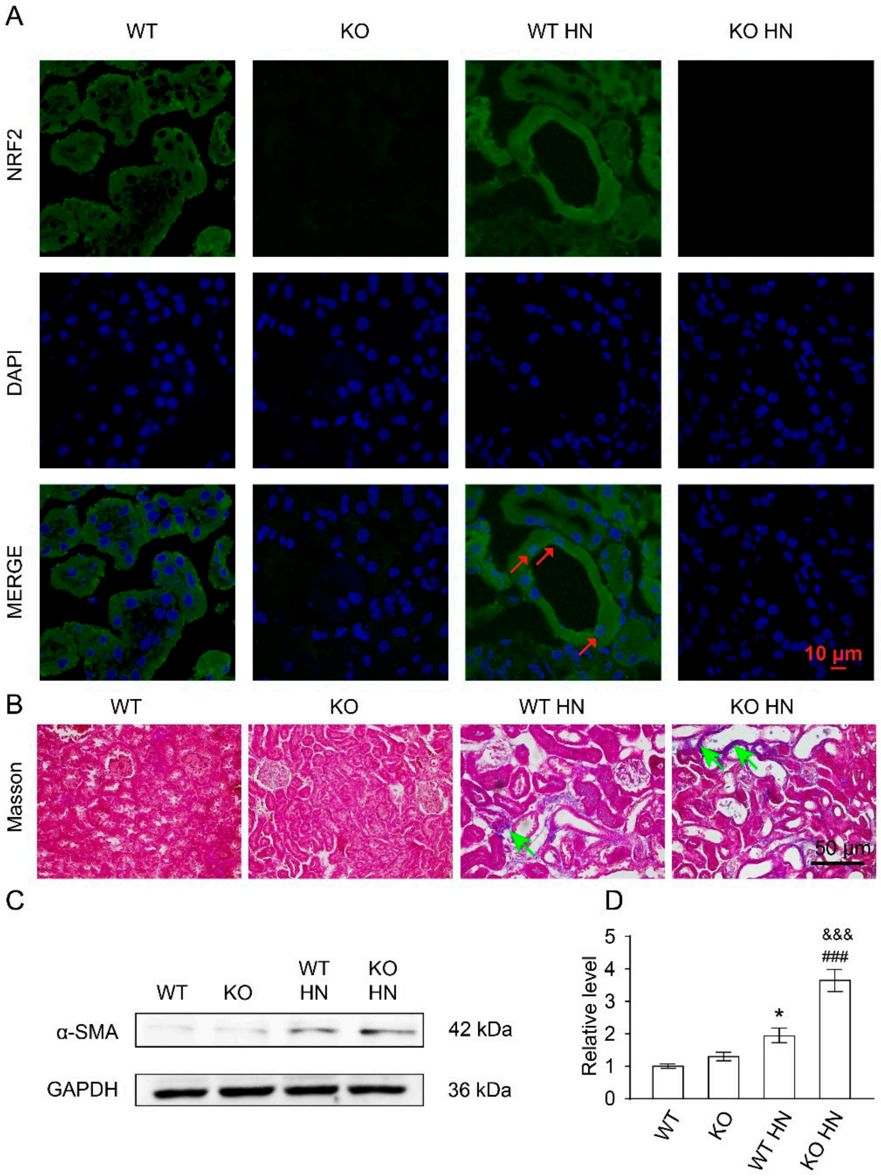

Figure S5 | (A) Immunofluorescence staining of NRF2 in kidneys in the indicated groups. Scale bar, 10 μm. (B) Masson staining of kidney tissue. Green arrows represent collagen deposits that are stained blue. Scale bar, 50 μm. (C) Western blot of α-SMA in the kidneys in the indicated groups. (D) Quantitative analysis of the expression of α-SMA. Data represent means ± SEM. $^{*}P < 0.05$ *vs.* wild type (WT) group. $^{\#\#\#}P < 0.001$ *vs.* *nrf2* knockout (KO) group, $^{\&\&\&}P < 0.001$ *vs.* wild-type hyperuricemia nephropathy (WT HN) group. Data are from kidneys of four mice.
